# Combination of electroacupuncture and pharmacological treatment improves insulin resistance in women with polycystic ovary syndrome: Double-blind randomized clinical trial

**DOI:** 10.18502/ijrm.v20i4.10900

**Published:** 2022-05-23

**Authors:** Raden Muharam, Ph.D., Adiningsih Srilestari, Hasan Mihardja, Lydwina Juvanni Callestya, Achmad Kemal Harzif

**Affiliations:** ^1^Department of Obstetrics and Gynecology, Faculty of Medicine, University of Indonesia, Jakarta, Indonesia.; ^2^Department of Medical Acupuncture, University of Indonesia, Jakarta, Indonesia.

**Keywords:** Acupuncture, PCOS, Insulin resistance, HOMA-IR.

## Abstract

**Background:**

Acupuncture is a nonpharmacological treatment which has been known to improve ovulatory function in polycystic ovary syndrome (PCOS) women. Acupuncture modulates the somatic and autonomic nervous systems, which regulate endocrine and metabolic functions to impact ovulatory functions.

**Objective:**

To investigate the effectiveness of electroacupuncture (EA) and pharmacological combination therapy on improving insulin resistance in women with PCOS.

**Materials and Methods:**

This double-blind, randomized clinical trial was performed on 44 participants from March to September 2018 at Cipto Mangunkusumo National hospital, Jakarta, Indonesia. Participants were randomly allocated to treatment (true EA + medication) and control group (sham EA + medication) in a 1:1 ratio using a web-based computer random-number generator. Randomization was carried out by an independent project manager. Both groups received 12 sessions of acupuncture therapy and metformin as pharmacological therapy.

**Results:**

This study showed a significant decrease in the Homeostatic Model Assessment for Insulin Resistance index in the true EA + medication group before and after therapy (p = 0.014).

**Conclusion:**

The combination of EA and pharmacological therapy effectively improves insulin sensitivity in women with PCOS.

## 1. Introduction

Polycystic ovary syndrome (PCOS) is one of the most well-known endocrine problems in women of reproductive age. It is described by persistent anovulation and hyperandrogenism, frequently connected with obesity and insulin resistance (1). PCOS should be considered a serious problem because of its implications for long-term health regardless of a woman's reproductive age. This needs to be seen as a condition that will last a lifetime, not just tied to pregnancy (2). Based on the Rotterdam consensus in 2004, a woman is diagnosed with PCOS if she has 2 of the following 3 conditions: oligo or anovulation; hyperandrogenism; and multi cystic ovaries (3).

PCOS affects all women regardless of race and nationality. This condition affects 7-10% of women worldwide. PCOS is the most common cause of infertility characterized by oligo-anovulation and insulin resistance, while hyperinsulinemia is found in 50-70% of women diagnosed with PCOS (4, 5). Women with PCOS are at high risk of diabetes, dyslipidemia, atherosclerosis, and cardiovascular problems. In addition, women with PCOS are at high risk for miscarriage, gestational diabetes, preeclampsia, and premature pregnancy (6, 7).

Based on current PCOS criteria (8), about 4-8% of women during reproductive years suffer from PCOS, and in the population of infertile women with anovulatory causes, 75% are due to PCOS (9). The prevalence of women with PCOS in reproductive age was 4.5%. This is due to the role of insulin resistance in the pathophysiology of PCOS, and the lifestyle of people causes the increase in insulin resistance with a high-calorie diet but with a sedentary lifestyle (10, 11). 60-80% women with PCOS are affected with metabolic issues (12). Data form the U.S shows 50-80% women are obese, 30-50% have impaired glucose tolerance, and 8-10% have type 2 diabetes mellitus (13). Several studies concluded that acupuncture had been shown to improve symptoms of PCOS and insulin resistance (14-16).

A study showed that repeated acupuncture therapy could result in improving the endocrine and metabolic function of PCOS women with obesity (16). A previous study also showed that acupuncture combined with electrical stimulation is effective in reducing diabetes-induced muscle atrophy. Acupuncture acts by mimics the effects of physical exercise in skeletal muscle and adipose tissue that leads to stimulate their bioactivity, hence activate regeneration (17). Research on the effect of acupuncture in PCOS women has never been done before in Indonesia.

This study investigates the effectiveness of electroacupuncture (EA) and pharmacological combination therapy on improving insulin resistance in PCOS women.

## 2. Materials and Methods 

### Study design

The study used a double-blind, randomized clinical trial with a control design. The trial was conducted from March 12 ${}^{\text{th}}$ to September 21 ${}^{\text{st}}$ 2018, at the Acupuncture Clinic and the Obstetrics-Gynaecology Clinic in Cipto Mangunkusumo National hospital (RSCM), Jakarta, Indonesia. This study included 44 participants divided into 2 groups.

### Population

Participants were recruited from women referred to the RSCM with PCOS diagnosis. PCOS was diagnosed by gynecologists. Participants were considered eligible upon meeting the following conditions: 1) 18-40 years old women; 2) body mass index ≥ 23 kg/m^2^; 3) volunteering to join this research and giving informed consent.

The exclusion criteria included: 1) Suffering from malignancy; 2) Diagnosed with endocrine disorders associated with steroid sex hormones such as congenital adrenal hyperplasia, Cushing's syndrome, and androgen-secreting tumor 3) Receiving pharmacological therapy within 12 weeks before recruitment; 4) Undergoing acupuncture therapy within 12 wk before recruitment; 5) Having absolute contraindications to EA therapy, such as heart rhythm disorders and women with pacemakers; 6) Currently in lactation.

Drop out criteria included: 1) Participants who did not complete 3 consecutive acupuncture sessions during the study period; 2) Participants who did not complete the study until the last blood sampling.

### Sample size

**Figure 1:**
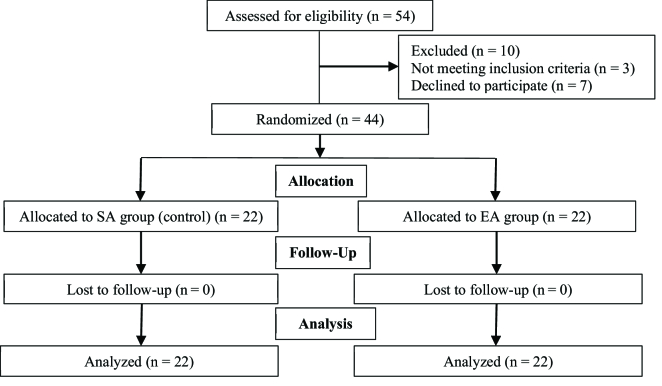
Study design.

Using the formula for 2-group randomized controlled trials (18). Based on the calculations using α (the significance level) = 0.05, β (the power of test) = 0.20 (80%), and using a previous study by Zheng et al as reference for population variance and smallest effect of interest (19), the minimal sample size needed was 22 participants per group. Participants were divided into 2 groups: 1) Treatment group treated with EA and metformin 2 × 500 mg; 2) Control group treated with sham EA and metformin 2 × 500 mg (Figure 1).

### Intervention protocol

In particular, manual and electrical stimulation of the acupuncture needle is generally comparable in achieving therapeutic benefit, with electrical stimulation mainly considered as a superior. Further, electrical stimulation is frequently preferred in basic science research because the stimulation parameters are easily measured in frequency, intensity, and duration.

The treatment group (EA group): before starting the first session of therapy and after the last acupuncture session, the patient fasted for 10-12 hr before taking blood samples. Blood sampling and reading of the results were carried out by RSCM laboratory analysis. The patient is lying, skin at points where EA will be performed and disinfected.

Puncture at the Zhongji (CV3), Guanyuan (CV4), Qihai (CV6), Tianshu (ST25), Shuidao (ST28), Zusanli (ST36), Chengsan (BL57), and bilateral Sanyinjiao (SP6) (Table I) with a continuous wave frequency of 2 Hz, the intensity was set based on the comfort of the patient, for 30 min. The EA needles (HUANQIU, Suzhou, China; size 0.25 x 40 mm) were connected to the SDZ-V nerve and muscle electrostimulator (Hwato, Jiangsu, China). Participants have to visit for acupuncture therapy 3 times per week, with a distance of 1-2 days, for 12 times. Pharmacological therapy used is metformin (Hexpharm Jaya, Jakarta, Indonesia) 2 × 500 mg per day.

The control group (Sham EA group) received the same procedure, however rather than EA, they had only needles with patches on the skin. The participants were blinded to which group they were assigned.

Randomization was done using a computer-based random block table randomizer. The authors used blocks with the size of 4, so 11 blocks were generated. Blocks were then randomly chosen to determine the participant's assignment into the groups. The randomization process was carried out by an independent project manager. Therefore, the executors, and evaluators were sufficiently blinded.

Initial examinations were carried out in the form of measuring body weight, waist circumference, height, fasting blood sugar levels, fasting insulin, and the Homeostatic Model Assessment for Insulin Resistance (HOMA-IR) index. Blood sampling and reading the results were carried out in the RSCM laboratory.

All participants who met the criteria were evenly allocated to the intervention group arm and control group using computer-generated blocked randomization. None of the participants dropped out.

**Table 1:** Locations of acupoints for treatment and control groups


** Acupoints**	**Location**
** Zhongji (CV3)**	Abdomen 4 cm below the umbilicus in the anterior median line
** Guanyuan (CV4)**	Abdomen 3 cm below the umbilicus in the anterior median line
** Qihai (CV6)**	Abdomen 1.5 cm below the umbilicus in the anterior median line
** Tianshu (ST25)**	Abdomen 2 cm lateral to the umbilicus
** Shuidao (ST28)**	Abdomen 3 cm below the umbilicus, 2 cm lateral to the anterior median line
** Zusanli (ST36)**	Anterior lower limb, on an imaginary line connecting the point of Dubi ST35 with Jiexi ST41, 3 cm below the Dubi ST35 point
** Chengsan (BL57)**	The posterior side of the lower leg, at the point, connecting the calcaneus tendon with the 2 heads of the gastrocnemius muscles
** Sanyinjiao (SP6)**	Posteromedial side of the lower limb tibia, 3 cm above the medial malleolus

### Ethical considerations

Signed informed consent was obtained from all of the participants. The study was approved by the Ethics Committee of the Faculty of Medicine, University of Indonesia, Jakarta, Indonesia (Code: 0248/UN2.F1/ETIK/2018).

### Statistical analysis

All samples who met the inclusion criteria were assessed for the fasting glucose, fasting insulin, and HOMA-IR index at the initial (1 ${}^{\text{st}}$ session) of acupuncture therapy and the end (12 ${}^{\text{th}}$ session). Therapeutic outcomes were assessed by comparing the mean scores of each measurement. Data normality test used Shapiro-Wilk. Statistical tests used for analysis depend on the variables.

For intra-group analysis, paired *t* test was used on the normally distributed variables, and Wilcoxon test on the not normally distributed ones. While for inter-groups analysis, Student's *t* test was used on the normally distributed variables, and the Mann-Whitney test on the not normally distributed ones. A p-value < 0.05 is statistically significant. Statistical analysis was conducted using SPSS version 20 (IBM, Armonk, NY, USA).

## 3. Results

This study was carried out on 44 PCOS women who met the inclusion criteria. All participants followed the study to completion.

### Research participants characteristics

An assessment of the research Participants' characteristics was conducted to identify potential bias causes in the results. The characteristics assessed were age, height, weight, BMI, waist circumference, initial fasting glucose, initial fasting insulin, and initial HOMA-IR. Statistical analysis showed no statistically significant difference in baseline characteristics between groups (Table II).

### Body mass index before and after intervention

This study assessed whether there is a correlation between the intervention received with BMI of participants in both groups before and after the intervention. Baseline data analysis showed no significant difference in BMI between groups (p = 0.52) (Table II).

Table III shows the mean BMI in the treatment and control groups. There were significant differences in the BMI before and after intervention in both groups. However, we found no significant difference when comparing the BMI after treatment between the 2 groups (p = 0.45).

### Fasting blood glucose levels before and after intervention

This study assessed whether there is a correlation between the intervention received with fasting blood glucose of participants in both groups before and after the intervention. Baseline data analysis showed no significant difference in fasting blood glucose levels between groups (p = 0.12) (Table II).

Table III shows the mean fasting blood glucose level in the treatment and control groups. Intragroup analysis showed no significant difference before and after treatment. We did not find any significant difference in the mean fasting blood glucose levels after treatment between groups (p = 0.12).

### Fasting insulin levels before and after intervention

Normality test showed that the participant's fasting insulin levels were not normally distributed therefore non-parametric tests were used. Baseline data analysis showed no significant difference in fasting insulin levels between groups (p = 0.26) (Table II).

Comparison of median fasting insulin levels after intervention between the 2 groups showed no significant difference (p = 0.54). However, it was found that the decrease in median fasting insulin levels from before to after intervention in the treatment group was statistically significant (p = 0.042) and higher (4.46 μIU/mL) compared to the control group (1.27 μIU/mL; p = 0.661) (Table III).

### HOMA-IR index before and after intervention

The normality test showed that the participant's HOMA-IR index data were not normally distributed therefore non-parametric tests were used for analysis. Baseline data analysis showed no significant difference in HOMA-IR index between groups (p = 0.19) (Table II).

Table III shows that when comparing before to after intervention, the decrease of median HOMA-IR index in the treatment group was 0.82. It was found to be statistically significant (p = 0.014). Meanwhile, on the contrary, the median HOMA-IR index in the control group increased by 0.09 points. Comparison of median HOMA-IR index after intervention between the 2 groups showed no significant difference (p = 0.35).

### Side effects incidence during the study

From 44 study Participants, 2 of them developed a hematoma.

**Table 2:** Research subject baseline characteristics (n = 22/each)


** Characteristics**	**Treatment group**	**Control group**	**P-value**
Age (yr)*	27.91 ± 4.09	28.14 ± 3.21	0.84
Body heights (cm)*	156.82 ± 4.99	157.05 ± 6.37	0.90
Body weight (kg)*	77.68 ± 16.55	74.55 ± 12.25	0.48
Body mass index (kg/m^2^)*	31.33 ± 5.86	30.27 ± 4.95	0.52
Waist circumference (cm)*	96.07 ± 11.37	95.87 ± 10.13	0.95
Initial fasting blood glucose (mg/dL)*	91.41 ± 10.63	85.82 ± 12.52	0.12
Initial fasting insulin**	20.28 (14.34)	16.68 (9.42)	0.26
Initial homeostatic model assessment for insulin resistance**	4.49 (3.73)	3.27 (1.88)	0.19
*Data presented as Mean ± Standard deviation, Student's* t* test. **Data presented as median (IQR), Mann-Whitney Test			

**Table 3:** Comparison of variable outcomes before and after intervention in study groups


** Variables **		**Treatment group**	**Control group**
Body mass index*			
	** Before**	31.32 ± 5.8686	30.27 ± 4.9530
	** After**	30.82 ± 5.9531	29.55 ± 5.0921
	** P-value**	< 0.001	< 0.001
Fasting blood glucose*			
	** Before**	91.41 ± 10.635	85.82 ± 12.519
	** After**	89.91 ± 8.326	85.18 ± 11.236
	** P-value**	0.261	0.496
Fasting insulin levels (days)**			
	** Before**	20.28 (14.34)	16.68 (9.42)
	** After**	15.82 (11.40)	15.41 (10.67)
	** P-value**	0.042	0.661
HOMA-IR index**			
	** Before**	4.49 (3.73)	3.27 (1.88)
	** After**	3.67 (3.28)	3.36 (2.52)
	** P-value**	0.014	0.592
*Data presented as Mean ± Standard deviation, Paired *t* test. **Data presented as median (IQR), Wilcoxon Test. HOMA-IR: Homeostatic model assessment for insulin resistance			

## 4. Discussion

The result of this study found that there were significant difference in the BMI before and after intervention in both groups (p < 0.001). Our study also found significant difference in fasting insulin levels (p = 0.042), and HOMA-IR index (p = 0.014) in treatment group before and after intervention.

Several previous studies conducted overseas stated that EA could help relieve metabolic syndrome in PCOS women. Besides, EA also provides almost no complications or side effects as in conventional management that already exists (20, 21).

PCOS is one of the most well-known endocrine problems in women of reproductive age and directly associated to insulin resistance. PCOS management aims to make the oligoovulation or anovulation cycle ovulate and alleviate metabolic syndrome.

The selection of points in this study is based on several previous studies that have proven effective in improving insulin resistance (22). Previous study assessed the effectiveness of acupuncture on the abdomen to look at endocrine and metabolic functions in obese PCOS women and compared it with metformin (19). In the study, CV4 Guanyuan, CV6 Qihai, CV12 Zhongwan, and CV13 Shangwan were used bilaterally at ST21 Liangmen, ST25 Tianshu, and ST28 Shuidao points, which are located near the stomach and close to the fat deposits. According to the baseline data scores and 6 months after that, fasting insulin, 2 hr post-prandial blood insulin, and HOMA-IR were significantly reduced in 2 groups (p < 0.05). This study concludes that acupuncture in the abdomen and metformin improve endocrine and metabolic function in obese women with PCOS. Another study conducted to assess the effect of acupuncture on weight loss. One of the points used is ST36 Zusanli and SP6 Sanyinjiao bilaterally. The levels of insulin, leptin, ghrelin, and cholecystokinin were checked by enzyme-linked immunosorbent assay. In the final results of the study, insulin levels decreased significantly (p < 0.05) compared to the control group and also affected the decrease in BMI (23).

In PCOS, there is evidence of dysregulation of the opioid system at the central and peripheral levels. In women with PCOS, there are low levels of β-endorphins which are associated with hyperinsulinemia. Low-frequency EA increases the low β-endorphin concentration, decreasing hyperinsulinemia and improving insulin sensitivity (24). In addition, EA facilitates the release of endogenous opioids in the central nervous system and into the circulation, resulting in the activation of specific opioid receptors. In diabetes, insulin secretion is enhanced by β-endorphin via activation of μ-receptors in the pancreas (25, 26).

A randomized controlled clinical trial in 39 women was conducted to compare the effect of combination metformin therapy with acupuncture and metformin monotherapy on insulin sensitivity in type II DM women. The treatment group received acupuncture with EA stimulation of 15 Hz, 10 mA for 20 min. The points used were ST25 Tianshu, SP15 Daheng, ST28 Shuidao, CV12 Zhongwan, and CV6 Qihai, and were given Sanjiao's ear points, hunger point, stomach, shenmen, endocrine, and spleen. The results of the study indicate that the combination of metformin and acupuncture significantly improves body weight, BMI, fasting blood sugar levels, insulin, HOMA-IR index, IL-6, TNF-α, leptin, adiponectin, GLP-1, resistin, serotonin, free fatty acids, TG, LDL, HDL, and ceramides. The conclusion of the study also proves that the combination of acupuncture and metformin therapy is more effective in increasing insulin sensitivity by reducing body weight and inflammation, and improving fat and adiponectin metabolism (27). A previous study also demonstrates that acupuncture effectively reduces BMI, so it is used for the treatment of obesity or overweight, especially in Asia (28).

A study involving 32 PCOS women randomized the participants to a treatment group, manual acupuncture or low-frequency EA, and a control group with physical therapy. Both are done 2 times a week but for 10-13 wk. Manual acupuncture and EA therapy in lean or obese women with PCOS increase ovulation frequency and improved insulin sensitivity (24). Result of the study found that insulin sensitivity values were higher in the acupuncture group than in the control group (p < 0.01). This study shows that one-time therapy and combining manual acupuncture and EA will improve glucose absorption throughout the body during and after stimulation in women with or without PCOS (29).

In PCOS rats that were insulin resistant with dihydrotestosterone induction, peripheral insulin sensitivity increased with low-frequency EA 3 times a week for 4-5 wk, and obtained normal results if performed 5 times per week (30). This normalization reflects an increase in Glucose Transporter type 4 translocation, including on the plasma membrane of muscle cells. EA low frequency also increases glucose uptake in skeletal muscle similar to that produced by physical exercise (31). Physical exercise increases β-endorphin secretion and insulin sensitivity by increasing post-receptors in the insulin signaling pathway, which corresponds to insulin receptor substrate 1 and phosphoinositide 3-kinase and makes enhanced glucose transporter type 4 translocation and glucose consumption better (32). EA increases insulin sensitivity through Sirtuin-1 regulation and peroxisome proliferator-activated receptor-γ coactivator-1α. Furthermore, EA increases peripheral blood flow and glucose uptake in skeletal muscles in response to muscle twitching reflexes during manual or electrical stimulation. In addition, there was a relationship between EA dose and response to increased insulin sensitivity (33).

This study showed an improvement in insulin sensitivity using a combination of metformin and acupuncture. Acupuncture groups showed significant changes compared to the metformin group in terms of decreased BMI, waist-hip ratio, and increased menstrual frequency (p < 0.05). In addition, acupuncture on the abdominal point and metformin improves endocrine and metabolic function in obese PCOS women with few side effects (19).

The side effect that occurred during the study was hematoma in 2 study Participants at the needle insertion site, which disappeared within 1 wk. There were no serious side effects such as infection. Thus, EA can be a safe and potential therapy to be combined with medical therapy, to improve symptoms of PCOS and reduce blood insulin levels.

The limitation of this study is that the diet and physical exercise of the research participants are not closely monitored, so there is a possibility of bias. In addition, the time of this research was short enough that the researcher could not make long-term observations. However, we believe this is the first study to investigate the effectiveness of EA and pharmacological treatment combination therapy on PCOS in Indonesia. Our methodology is also better designed compared to previous similar studies. We hope this study can pave the way for further studies in this field.

## 5. Conclusion

The combination of EA and pharmacological therapy is proven effective for insulin sensitivity improvement in PCOS women.

##  Conflict of Interest

The authors declare to have not no conflict of interest.
